# Multilayer Thickness Measurements below the Rayleigh Limit Using FMCW Millimeter and Terahertz Waves

**DOI:** 10.3390/s19183910

**Published:** 2019-09-11

**Authors:** Nina S. Schreiner, Wolfgang Sauer-Greff, Ralph Urbansky, Georg von Freymann, Fabian Friederich

**Affiliations:** 1 Center for Materials Characterization and Testing, Fraunhofer Institute for Industrial Mathematics ITWM, 67663 Kaiserslautern, Germany; georg.von.freymann@itwm.fraunhofer.de; 2 Institute of Communications Engineering, Technische Universität Kaiserslautern, 67663 Kaiserslautern, Germany; sauer@eit.uni-kl.de (W.S.-G.); urbansky@eit.uni-kl.de (R.U.); 3 Department of Physics and Research Center OPTIMAS, Technische Universität Kaiserslautern, 67663 Kaiserslautern, Germany

**Keywords:** FMCW, thickness measurement, Rayleigh limit, millimeter-wave, terahertz, non-destructive testing, high accuracy, multiple reflections

## Abstract

We present thickness measurements with millimeter and terahertz waves using frequency-modulated continuous-wave (FMCW) sensors. In contrast to terahertz time-domain spectroscopy (TDS), our FMCW systems provide a higher penetration depth and measurement rates of several kilohertz at frequency modulation bandwidths of up to 175 GHz. In order to resolve thicknesses below the Rayleigh resolution limit given by the modulation bandwidth, we employed a model-based signal processing technique. Within this contribution, we analyzed the influence of multiple reflections adapting a modified transfer matrix method. Based on a brute force optimization, we processed the models and compared them with the measured signal in parallel on a graphics processing unit, which allows fast calculations in less than 1 s. TDS measurements were used for the validation of our results on industrial samples. Finally, we present results obtained with reduced frequency modulation bandwidths, opening the window to future miniaturization based on monolithic microwave integrated circuit (MMIC) radar units.

## 1. Introduction

Millimeter and terahertz wave technology provides a non-destructive and contact-free method for accurate thickness measurements of dielectric multilayer structures. Alternative technologies such as micrometer gauges or microscopy methods require direct access to the cross-section of the layer structure, which is often not easily accessible due to the sample geometry. For non-destructive measurements, it is necessary to employ technologies that can penetrate the individual layers from the outside of the sample. Among well-established non-destructive testing methods for thickness determination, solely ultrasound [1] and optical interferometric [2] techniques determine layer thicknesses down to a few micrometers in dielectric multilayer structures, although the latter can only be applied for optically transparent materials. Ultrasound techniques often require a coupling medium and are thus not contact-free. Moreover, the sample materials are required to be of low acoustic attenuation. In contrast to these technologies, broadband terahertz time-domain spectroscopy (TDS) systems have proven to be highly suitable for non-destructive and contact-free layer thickness inspection of dielectrics, such as multilayer car paints [3,4,5,6] and pharmaceutical products [7] with individual layer thicknesses down to 5 µm [3]. Frequency-dependent attenuation of the sample under test may limit the useable bandwidth below the capability of the device of 4 THz [8,9], restricting the maximum thickness to typically a few millimeters.

Among a wide variety of measurement techniques for determining a material’s electromagnetic properties [10] in the microwave and terahertz spectrum, free-space methods such as those described in [11,12,13,14,15] can also be used to calculate the thickness of a material, since its frequency-dependent reflection and transmission coefficients are functions of permittivity, permeability, and thickness. The measurement setups in [11,12,13,14] require both reflection and transmission measurements using a vector network analyzer (VNA). These approaches have the potential to determine sub-micrometer material thicknesses of a single layer and can be applied to measure single-layer thicknesses of multilayer structures [14]. However, transmission measurements require two-sided access to the sample, which limits the application of these setups for quality control in industry. The authors in [15] present a method, which can be applied for layer thickness measurements of multilayer structures (solely) in reflection geometry and hence with single-side access and a single sensor unit. This is achieved by obtaining measurements over different angles of incidence or over a certain frequency range. In [15], the minimum thicknesses of the single layers have to be at least in the range of the wavelength.

An alternative to VNA measurements with discrete frequency steps are millimeter-wave frequency-modulated continuous-wave (FMCW) radar measurements. Such an approach was presented recently for the characterization of single-layer dielectrics in [16]. Especially concerning industrial applications, millimeter-wave and terahertz FMCW transceivers can provide kilohertz measurement rates at a high level of integration [17]. The inherent resolution limit of FMCW radars is determined by the bandwidth of the sensor and the refractive index of the layers. For example, with the bandwidths (40 GHz to 90 GHz) of the FMCW systems presented in [17], thicknesses of a few millimeters and below can be resolved.

Within this contribution, we focused on the industrial application of the FMCW technique for layer thickness determinations of multilayer systems. We used a model-based signal processing technique to overcome the inherent lower resolution limit [18] with a priori information such as the number of individual layers and a reference measurement for evaluating the material parameters. Similar to the approaches in [3,14,15], we fitted the calculated signals resulting from propagation models of the sample under test to the measured ones. In contrast to model-based frequency estimation algorithms such as in [19], our signal model enables the consideration of wave propagation aspects such as multiple reflections, which can be significant for the thickness measurements depending on the material parameters. To avoid possible variations in the measurement results, we used brute force optimization instead of more advanced optimization algorithms as in [14,15,16]. In industrial production environments, the material density may vary. Therefore, we primarily evaluated the optical path length difference of the layer structure, which in many cases provides sufficient information for process and quality control. In order to obtain physical thicknesses, we additionally used reference measurements on homogeneous and planar material samples to estimate the refractive indices of the different layers. We validated our results using accurately defined calibration samples and measurements of industrial multilayer products performed with a TDS setup. Finally, we addressed measurements with reduced modulation bandwidths for the potential use of monolithic microwave integrated circuit (MMIC)-based integrated FMCW radars [20,21,22].

## 2. FMCW Thickness Measurements

**Figure 1:**
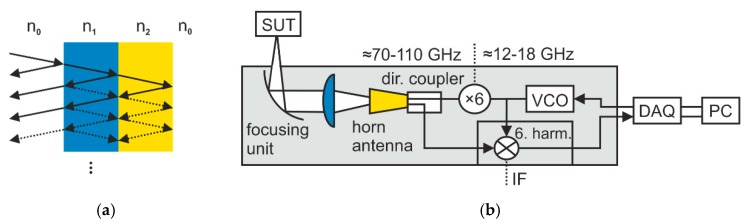
(**a**) Main (solid lines) and multiple reflections (dashed lines) within a multilayer sample under test (SUT) (**b**) Schematic of the measurement setup. VCO, voltage-controlled oscillator; DAQ, data acquisition unit; PC, personal computer; dir., directional; 6. harm., 6-th harmonic; IF, intermediate frequency.

We obtained the measurement results of this work with an all-electronic terahertz unit working in a monostatic transceiver configuration and employing the FMCW technique. A voltage ramp drove a voltage-controlled oscillator (VCO) resulting in a linearly frequency-modulated, continuous-wave oscillation described in its analytic representation as in [23]:

(1) $$s_{trans}\left(t\right)\;=\;e^{j2\pi F_{1}t+j\pi \frac{B}{T}t^{2}},$$

where *T* is the duration of the modulation cycle, *F*_1_ is the start frequency of the modulation, *t* is time, *B* is the bandwidth, and j is the imaginary unit. The signal was transmitted by a wave guide-coupled horn antenna of our measurement head to a sample under test (SUT) and then partly reflected by the different layer interfaces within the sample. Besides the main reflections, multiple reflections between the interfaces reached the antenna, as shown in Figure 1a. The detected signal is the sum of time delayed and attenuated portions of the transmitted one, as follows:

(2) $$s_{refl}\left(t\right)\;=\;\overset{K}{\underset{k=0}{\sum }}a_{k}e^{j2\pi F_{1}\left(t-\tau_{k}\right)+j\pi \frac{B}{T}{\left(t-\tau_{k}\right)}^{2}},$$

where $a_{k}$ is the amplitude of the *k*-th reflection, which depends on the refractive index of the layers. The number of total reflections K is infinite in theory for a single (or multilayer) system. However, due to the attenuation, the sum can be truncated after a finite number of terms. The received sum of reflections is mixed with the emitted signal. The digitized intermediate frequency (IF) signal is processed, which results in a sum of oscillations [23], as follows:

(3) $$s_{IF}\left[n\right]\;=\;\overset{K}{\underset{k=0}{\sum }}a_{k}e^{j2\pi F_{1}\tau_{k}+j\pi \frac{B}{T}\cdot \left(\frac{2n}{f_{s}}-{\tau_{k}}^{2}\right)}\sim \overset{K}{\underset{k=0}{\sum }}a_{k}e^{j2\pi f_{b}\frac{n}{f_{s}}+j2\pi F_{1}\tau_{k}},$$

where $f_{b}\left(\tau_{k}\right)\;=\;\frac{B}{T}\tau_{k}$ represents the characteristic beat frequencies, $\Phi \left(\tau_{k}\right)\sim 2\pi F_{1}\tau_{k}$ represents the zero phases, *n* is digital time, and $f_{s}$ is the sampling frequency. Therefore, the beat frequency can be used to determine the time delays and hence the layer distances and thicknesses. Our FMCW systems can provide operation frequencies up to 500 GHz with a frequency-modulation bandwidth of 175 GHz. As an example for our FMCW thickness measurement setup, Figure 1b depicts a schematic of a 100 GHz measurement system. A voltage-controlled oscillator (VCO) is driven by the output of a digital-to-analog converter of a data acquisition unit (DAQ, model PCI-6115 from National Instruments, Austin, TX, USA) to generate an FMCW oscillation with a modulation range from 11.8 GHz to 18.2 GHz. We assumed a linear frequency modulation for our model-based signal processing approach and compensated for nonlinearities of the voltage to frequency relation of the VCO by predistortion [24]. The output drives both, an active multiplier chain and a harmonic mixing receiver, which are attached to a directional coupler with a transmitting and receiving antenna. We used a combination of a polytetrafluoroethylene (Teflon) lens and an off-axis parabolic mirror to collimate and focus the emitted signal into the SUT at normal incidence. The signal reflections were guided back into the transceiver by the same optical setup. Mixing the received signal with the 6th harmonic of the VCO signal within the receiver generates an IF signal at its output, which is sampled by the DAQ’s analog-to-digital converter. Once the sensor was in thermal equilibrium (typically within 10 to 15 min), we performed a two-term calibration according to [25]. As long as the environment conditions do not change dramatically, the calibration remains efficient, since small temperature drifts do not significantly affect the measurements.

To evaluate the beat frequencies of the IF signal, we transformed the measured signal to the frequency domain by applying a Fourier transform. Due to the limitation of the measurement interval, which equals the duration of the modulation, the frequency spectrum of a single reflector corresponds to a sinc function with center frequency $f_{b}$. The distance between the object and the transceiver linearly depends on $f_{b}$, and can be accurately evaluated [21,26]. However, for thickness determination, the width of one main lobe restricts the ability to distinguish it from a second reflection peak and therefore limits the resolution. The distance between the maximum of one peak and the first zero crossing is defined as the Rayleigh resolution limit [17], as follows:

(4) $$\Delta r_{Ray}\;=\;\frac{c_{0}}{2Bn_{\mathrm{Medium}}},$$

where $c_{0}$ is the speed of light. This limit is given by the frequency modulation bandwidth and the refractive index of the propagated medium $n_{\mathrm{Medium}}$. For an estimate of the index, we evaluated a measurement of a well-defined object. Due to the relations given in Equation (4), the precision of that measurement effects the precision of the thickness measurement.

**Figure 2:**
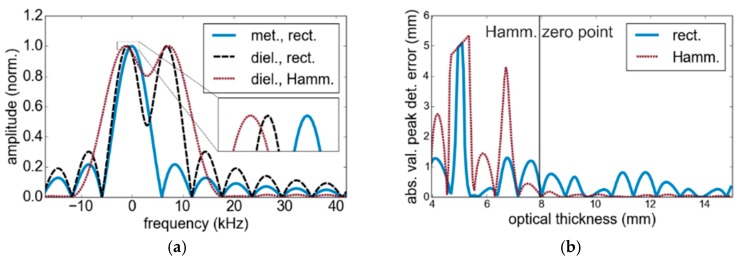
**(a)** Fourier amplitude spectrum of a single metal reflector (met.) in the calibration plane and a combination of two dielectric reflectors (diel.) with (Hamm.) and without (rect.) the application of a Hamming window function. (**b**) Peak detection error using a rectangular (blue) and a Hamming (red) window function.

Figure 2a illustrates simulation results, which indicate the shift of the main lobe for the case of an additional reflecting surface. The zero frequency corresponds to the calibration plane and the exact position of first reflectors in all cases. However, sinc functions of additional reflections influence the first peak position either by the width of the main lobe toward positive or negative frequencies (as depicted in Figure 2a) or interfere with the side lobe. Since $f_{b}$ is proportional to the optical path length, the *x*-axis can be linearly transformed to represent range (distance) information. Even though the application of a Hamming window (Hamm.) reduces the side lobes, it doubles the width of the main lobe and correspondingly degrades the resolution. Figure 2b describes the thickness measurement error as a function of the thickness for a modulation bandwidth of 38 GHz. A peak detection algorithm is used to process the distance between the two highest local maxima. To avoid the processing of side lobes as maxima, we set a threshold of 0.5 times the maximum value. If we detected only one peak, then the resulting thickness was defined as zero. Only for thicknesses that exceed the distance to the first minimum after applying a Hamming window, an error below one percent of the optical path length difference can be achieved. As Figure 2b reveals, even thicknesses larger than the Rayleigh limit are processed inaccurately by peak detection, due to the influence of the side lobes. Hence, this limit does not describe the minimum thickness, which can be determined without bias. In order to obtain accurate measurement results, we developed a model-based signal processing approach, which considers interfering signal portions.

## 3. Model-Based Signal Processing Approach

**Figure 3:**
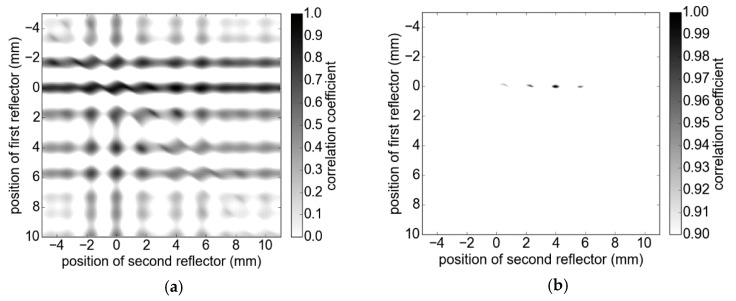
Solution space of the correlation approach for a single layer. The resulting thickness is calculated as the difference of *x*- and *y*-values of the maximum. From (**a**) to (**b**), the visibility is enhanced by adjustment of the displayed range of the correlation coefficient (the color version is provided as Supplementary Materials).

To resolve thicknesses close to the Rayleigh resolution limit and even below, a promising method is the correlation coefficient-based comparison of measurement signals and signal models, which represent the material structure of the SUT [18]. The highest correlation indicates the best approximation of the real positions and hence the evaluated thicknesses. Considering the simulated measurement scenario of two boundary surfaces in Figure 2a and given the assumption of only one main reflection per boundary surface, we calculated models for all potential positions of reflectors (brute force) within the correlation interval using an appropriate step size and compared them with the measured signal. Figure 3 depicts the solution space featuring numerous local maxima besides the global one. In order to achieve accurate results, a small step size of 25 µm was used. The amplitudes are given as $a_{2}\;=\;0.5a_{1}$ and additional influences such as spurious echoes and the phase noise are not included. The global maximum of the correlation coefficient of 1 accurately represents the thickness of 4 mm, as can be observed in Figure 3b. In many applications, a reasonable prediction of the correlation interval can be given, which can significantly reduce computation time. However, the complexity increases exponentially for every additional material layer. Since the distance of a SUT to the measurement system may vary during the inspection, large correlation intervals may be required to compensate for positioning errors. An alternative approach is the dynamic adaptation of the correlation interval’s position. We used the position of the first peak as a rough estimate of the position of the first reflection and accordingly shifted the correlation intervals. This significantly reduced the computational load.

Depending on the characteristics of the SUT, that is the dielectric constants and absorption coefficients of the materials as well as the layer thicknesses compared to the Rayleigh resolution limit, multiple reflections between boundary surfaces may have to be considered to obtain accurate results. For thicker and highly absorbing materials, multiple reflections are strongly attenuated and can be omitted for the signal processing due to negligible amplitudes. For a single layer, the amplitude ratio between the first multiple reflection of the second main reflection is as follows [27]:

(5) $$\frac{A\left(\mathrm{first}\;\mathrm{mupltiple}\;\mathrm{reflection}\right)}{A\left(\mathrm{second}\;\mathrm{main}\;\mathrm{reflection}\right)}\;=\;\frac{1-n_{1}}{n_{1}}\;\frac{n_{2}-n_{1}}{n_{2}n_{1}}.$$

**Figure 4:**
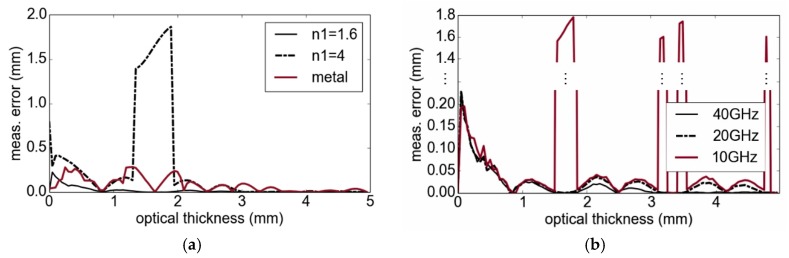
Thickness measurement (meas.) errors caused by disregarding multiple reflections: (**a**) dependency on refractive indices and (**b**) reduced bandwidth from 40 GHz to 10 GHz with a constant center frequency of 90 GHz.

In order to investigate the influence of multiple reflections on thickness measurements, we performed model-based calculations of the measurement error as a function of the optical path length difference. A bandwidth of 38 GHz and a correlation interval between 0 and 7 mm with a step size of 1 µm were used. Figure 4a shows the measurement error for three different cases of a simulated single layer: $n_{1}=1.6$ and air, $n_{1}=4$ and air, as well as $n_{1}=1.6$ and metal plate. For the first case, the error is less than one percent for an optical path length difference larger than 1.5 mm. Using $n_{1}=4$, and for thicknesses between 1.4 and 2 mm, the interference is significant and only one peak results from the processing. In Figure 4b, different modulation bandwidths for measurements of a layer with $n_{1}=1.6$ are considered. As the results indicate, the error increases with a lower bandwidth, due to the widened peaks. Because of the calculation results in Figure 4, for most cases, multiple reflections have to be considered in our signal model in order to obtain highly accurate measurement results (but in particular for high contrasts of refractive indices).

For multilayer materials, multiple reflections can interfere with the main reflection of any layer interface and different amplitude ratios are possible.

**Figure 5:**
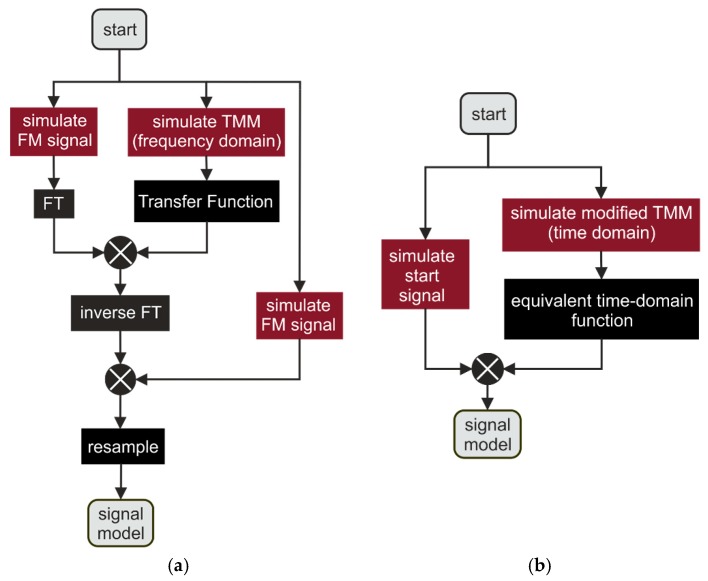
(**a**) Block diagram of the classical transfer matrix method (TMM) calculation. (**b**) Block diagram of the modified TMM calculation. FT, Fourier transformation.

Wave propagation can be implemented using recursive approaches such as ray tracing (infinite sum of reflections) [28] or the Rouard method [3]. In contrast to these, our approach uses the iterative transfer matrix method (TMM) [29,30]. It is based on matrix multiplication, does not depend on intermediate data, and hence provides more flexibility for a fast graphics processing unit (GPU) implementation. Figure 5a shows a block diagram of the classical TMM calculation approach adapted to our measurement setup. A cycle of the frequency-modulated (FM) signal is simulated considering the Nyquist theorem, which corresponds to several tens of millions of time samples. The signal is then transformed into the frequency domain and multiplied with a transfer function resulting from a large transfer matrix. Afterward, the inverse Fourier transformation of the product and a time-shifted FM signal are mixed. To obtain the resulting measurement signal, the product is downsampled according to our sampling rate. The block diagram in Figure 5b illustrates our modified TMM approach which is derived in [31] in detail. In contrast to [29,30], an even more efficient calculation of the signal model is achieved by only calculating the matrix of the samples of the measurement signal. Here, the simulated FM signal is multiplied with a much smaller equivalent time-domain function resulting from the modified transfer matrix. This method is justified due to the conservation of the amplitudes of the reflections within the mixing process (comparison of Equations (2) and (3)) and the transformation of the reflections’ time delay $\tau_{k}$ into a frequency and zero phase shift.

Similar to the classical TMM, our modification calculates a crossover matrix for each layer interface and a propagation matrix for each single layer. We assumed the materials to be non-dispersive for convenience. For an SUT with a total of *L* layers, the crossover matrix of the *l*-th to the *l*+1-th layer results in the following:

(6) $$D_{l,l+1}\left[n\right]=\frac{1}{t_{l,l+1}}\;\left(\begin{array}{cc} 1 & r_{l,l+1}\\ r_{l,l+1} & 1 \end{array}\right),$$

where $t_{l,l+1}$ represents the transmission and $r_{l,l+1}$ represents the reflection coefficients. The 0-th and the *l*+1-th layer correspond to air and the first layer is positioned in the calibration plane. The modified propagation matrix results in the following:

(7) $$P_{l+1}\left[n\right]\;=\;\left(\begin{array}{cc} e^{-j2\pi \frac{B}{T}\frac{n}{f_{s}}\Delta \tau_{l}-j2\pi F_{1}\Delta \tau_{l}} & 0\\ 0 & e^{j2\pi \frac{B}{T}\frac{n}{f_{s}}\Delta \tau_{l}+j2\pi F_{1}\Delta \tau_{l}} \end{array}\right),$$

where $\Delta \tau_{l}$ is the time delay within the l-th layer. The matrix is based on the beat frequency and phase shift caused by changing the distance between measurement head and the SUT, which can be observed by comparing Equations (2) and (3). Using Equations (6) and (7), the combined matrix of the l-th layer results in $M_{l+1}\left[n\right]\;=\;D_{l,l+1}\left[n\right]*P_{l+1}\left[n\right]$. To calculate the complete transfer matrix M[n], the matrices of the single layers are multiplied, and hence the equivalent time-domain function $h_{\mathrm{equi}}\left[n\right]$ = $M_{10}\left[n\right]/M_{00}\left[n\right]$ is multiplied with the simulated measurement signal [31].

In the following section, some measurement examples are given, which illustrate the difference before and after our implementation of the TMM into our signal model.

## 4. Measurement Results

We validated our signal processing approach by test measurements using a sensor operating at 90 GHz center frequency with a modulation bandwidth of 39.5 GHz (which differs from 38 GHz in the simulation section) and a measurement rate of 5 kHz. The corresponding optical resolution limit was 3.8 mm. In the first subsection, we addressed single-layer calibration plates, which validate our model-based approach. We then discussed measurement results of multilayer plastic pipes representing an emerging industrial application for millimeter-wave FMCW radars [32]. Since the layer thicknesses vary throughout the samples, we compared our results with terahertz time-domain spectroscopy (TDS) measurements. Finally, we addressed the potential application of our approach with reduced frequency modulation bandwidths. The latter provides an interesting option for industrial applications, since FMCW radars with relatively small bandwidths are readily available as MMIC modules.

### 4.1. Calibration Plates

In a first step, we performed measurements on well-defined calibration plates with constant thicknesses—one consisting of Pertinax and one of acrylic, as shown in Table 1. We performed thickness evaluation using two processing methods, namely, the modified TMM, which describes the complete wave propagation, and the basic correlation method (BC), which includes only the main reflections. We compared the measurement results and model-based calculation outputs in both cases. The calculations were performed in steps of 1 µm within the intervals given in Table 1. To estimate the refractive indices, we first measured the optical path length differences of the single samples and used the thickness specifications of the individual samples to evaluate the refractive indices and corresponding Rayleigh limits. The Pertinax sample and the acrylic sample were specified with a thickness of 8.04 mm ± 1% and 1.5166 mm ± 0.4%, respectively. Next, we stacked both plates to a multilayer sample and inspected it with our measurement system. We positioned the combined samples such that the Pertinax plate faced the FMCW transceiver and strongly absorbed the incoming radiation. We used a step size of 5 µm for the signal model calculations in the evaluation of the measurement data. The results are shown in Table 2, indicating that we were able to measure both layers accurately. Moreover, the standard deviation of 10 measurements was lower than 2.6 µm for the BC and 2.2 µm for the TMM. However, these values may be influenced by the chosen step size. For the TMM, the standard deviation of acrylic even resulted in 0 µm and was therefore re-evaluated for a step size of 2.5 µm, resulting in a standard deviation of 0.8 µm. Besides the higher precision, TMM delivered results that are slightly more accurate.

In a second measurement scenario, the thickness of the single acrylic plate was measured, while a metal tape covered the back of the sample. For data processing, the evaluated refractive indices of the different algorithms were reused. The correlation interval was from 0 to 2 mm and the step size was 3.1 µm. While the TMM accurately reproduced the thickness with a value of 1.53 mm, the BC approach yielded a thickness of 0.5 mm and therefore resulted in a high deviation due to the increased influence of the multiple reflections according to Section 3.

To investigate the potential for measurements of thin single layers, 13 individual calibration foils made of biaxially-oriented polyethylene terephthalate with thicknesses between 72.7 and 291 µm were inspected in a previous study [33]. The results showed a deviation of less than 5% down to a layer thickness of approximately 121 µm, which corresponds to roughly 6% of the Rayleigh resolution limit. Further investigations concerning the precision estimated as the standard deviation of the FMCW approach are addressed in [34].

### 4.2. Industrial Multilayer Structures

**Figure 6:**
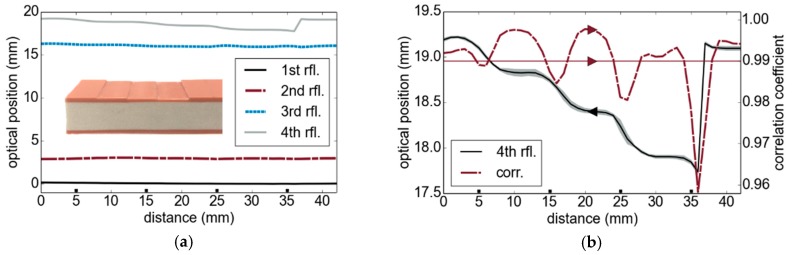
Multilayer tube section: (**a**) optical positions (path length difference) of reflectors, (inset) photograph of the stepped multilayer section; (**b**) increased depiction of the stepped layer and the corresponding correlation coefficient (corr.) which is related to the right *y*-axis; the squares along the *x*-axis indicate the step positions. rfl., reflection.

In a next step, we performed thickness measurements on industrial multilayer structures. The inset in Figure 6a depicts a cross-sectional view of a multilayer tube wall. It consists of two outer polyvinyl chloride (PVC) layers and a thick internal recyclate foam. A step wedge with approximately 300 µm step height and 1 cm step width was milled into the top outer layer. The SUT was measured from the side of the flat surface (bottom layer in Figure 6a), so that the layer with the step wedge represented the last layer of the structure. The average determined thicknesses from five repetitive measurements are depicted in Figure 6a. Figure 6b shows a magnification of the 4th reflector’s position and reveals the stepped shape of the tube sample’s back surface. The gray surrounding of the curve represents the standard deviation as the square root of the unbiased variance of five measurements. Due to the finite diameter of the terahertz beam (≈5 mm spot size), the edges of the steps appear as smooth transitions. The dashed line corresponds to the correlation coefficient from our signal model approach. While the signal model could potentially be further improved by more accurate information on the measurement setup and the material parameters, a coefficient larger than 0.99 already guarantees a high correlation. Besides the system parameters, further aspects such as the optical configuration will be considered in future works in order to improve the measurement results. Note that a comparative TDS thickness measurement was not possible for this specific sample due to a high absorption within the sample (considering the full spectrum) and the large sample thickness of roughly 1.3 cm exceeding the fast sampling range window of our TDS setup.

### 4.3. Verification by Comparison with Terahertz Time-Domain Spectroscopy Measurements

**Figure 7:**
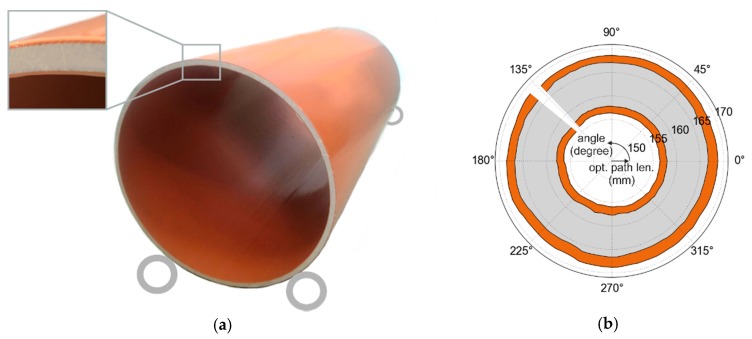
(**a**) Photograph of the SUT consisting of three layers including a schematic of the two pairs of wheels. (**b**) A mechanical measurement enables the conversion of the previous results to the optical path length (opt. path len.) to the ellipse center. The outer surface corresponds to the first reflection in Figure 8a.

In order to verify our measurement approach and to prove its robustness against varying sample positions, we measured the wall thickness of a plastic tube along its circumference and compared the results to TDS measurements of the same sample in reflection geometry (≈1 mm focal spot size in air). The curvature of the sample itself as well as the different focal spot size of both measurement systems were not considered in the evaluation. The tube wall had a similar layer structure as the one discussed in Section 4.2 (Figure 6a)—without the additional steps on the back surface—but was significantly thinner. As schematically shown in the photograph in Figure 7a, the tube was placed on two pairs of wheels for rotation. In the case of the FMCW measurements, the sensor was positioned beneath the tube and pointing toward the tube wall perpendicular to the surface. Since the sample exhibited some eccentricity, the distance between wall and FMCW sensor varied significantly during tube rotation. For Figure 7b, we therefore used a concentricity gage to map the distance between the measured reflections and the center of the tube.

**Figure 8:**
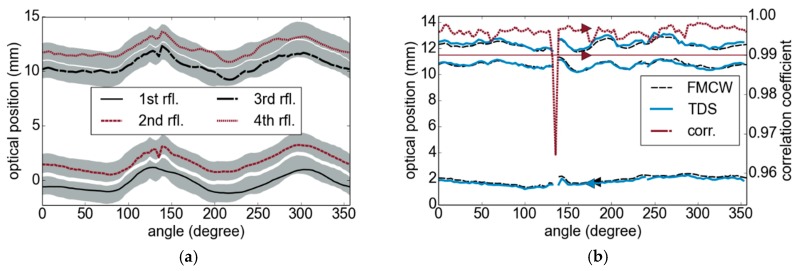
(**a**) Optical positions (optical path length differences) of the reflecting boundary surface. (**b**) Comparison of time-domain spectroscopy (TDS) and frequency-modulated continuous-wave (FMCW) measurements: the first boundary surface is used as the reference plane to adjust the values.

For the TDS measurements, the sensor was positioned above the tube and its distance was adjusted manually, in order to keep the sample within the measurement window of the fast sampling range of the TDS system. For the FMCW measurements, we calculated the first peak of the Fourier spectrum as an estimate to adapt the interval position, as indicated in Figure 8b by the gray areas surrounding the measured positions of the reflections. The variations of the first reflection can be estimated as 1.5 mm. Figure 8b, which compares the results of both techniques, shows an excellent agreement of the determined layer thicknesses. For a rotation angle of 135°, we could not obtain a clear result, which is possibly related to a seam in the outer layer of the tube wall. The measurements were taken manually in steps of 1 cm around the tube, which introduced slight deviations to the measurements by positioning errors. The TDS setup is characterized by a measurement time of 1 s per point (the FMCW measurement time was 200 µs) by a bandwidth of up to 4 THz using a Rouard method-based signal processing algorithm [3].

### 4.4. Toward Reduced Bandwidths and Highly Integrated Systems

**Figure 9:**
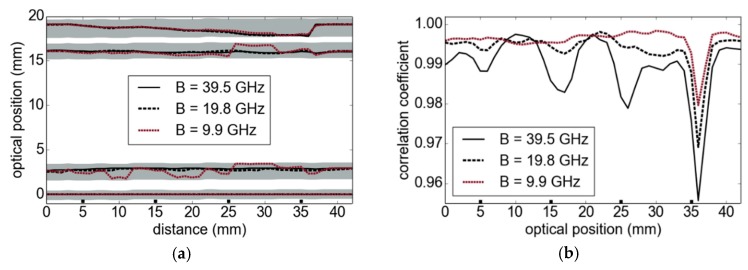
Stepped tube section: (**a**) the number of samples and, hence, the bandwidth is reduced; (**b**) correlation coefficient for the 4th reflection in dependence of the modulation bandwidth.

The increasing availability of highly integrated millimeter-wave radar sensors based on MMICs with considerably large frequency modulation bandwidths provides a high potential for widespread application of the underlying thickness measurement technique. While the full available bandwidth of 39.5 GHz and measurement rate of our sensor is still superior, we deliberately reduced the processed bandwidth in an additional measurement scenario with the same sample as introduced in Section 4.2. Figure 9 shows the results obtained with different reduced FMCW bandwidths. Even 19.8 GHz is sufficient to resolve the layers of the structure in Figure 6a very accurately. In contrast, the results with an even smaller bandwidth of only 9.9 GHz show strong deviations, in particular for the second and third boundaries. The gray areas in Figure 9a correspond to the correlation intervals of our signal processing approach. As indicated in Figure 9b, the correlation coefficient also becomes less sensitive to the stepped structure at lower modulation bandwidths. We expect to obtain better results by considering the optical aspects of the measurement setup and a better estimation of the material parameters, which have not been accessible as single layers. However, as part of our future work, we want to evaluate the material parameter using measurements of multilayer composition as done in [15].

## 5. Conclusions

Within this contribution, we presented a measurement approach for the inspection of multilayer dielectric samples such as PVC tubes. In contrast to well-established non-destructive testing techniques, the millimeter-wave and terahertz technology allows contact-free inspection of optically opaque, dielectric materials. While broadband terahertz TDS systems can resolve multilayer thicknesses down to several micrometers, they are often limited to thinner samples; the presented all-electronic FMCW approach benefits from the high penetration depth of millimeter and low-frequency terahertz waves in connection with superior performance at sub-millisecond measurement times. The inherent thickness resolution is limited by the measurement system’s bandwidth and its pre-evaluated refractive index but is overcome by a correlation-based signal processing approach, which compares the measured signal with the simulation result of an appropriate signal model. In this way, layer thicknesses down to roughly 6% of the Rayleigh resolution limit [33] and up to several centimeters of thickness can be determined. By analyzing the influence of multiple reflections on the accuracy, we found increasing deviations between the physical and measured thicknesses with increasing contrast of the refractive indices between adjacent layers. To counter this shortcoming, we included an optimized modification of the iterative TMM to our model, which describes the effects of complete wave propagation and enables an efficient implementation on graphics processing units. While we use simple brute force optimizations for our calculations, the implementation of more advanced optimization algorithms, such as in [14,15,16], can reduce the calculation time even further. We verified the suitability of our approach by validating the obtained measurement results with a set of well-defined calibration plates. In addition, we performed comparative TDS measurements on industrial tube walls for verification. Our FMCW measurement system operates at high measurement rates of approximately 5 kHz and thus provides inline inspection capabilities. The widespread commercial availability of FMCW radar sensors as MMICs allows a high-level of integration, for example, for compact and lightweight sensor systems. While the modulation bandwidth of such MMICs is still significantly lower than in our all-electronic sensor design, we demonstrated that our signal processing approach can potentially compensate for this limitation.

## Figures and Tables

**Table 1:** Parameters for the evaluation of the refractive index.

Material	Step Size (µm)	Optical Correlation Interval (mm)	Refractive Index BC	Refractive Index TMM	Rayleigh Limit
Pertinax	1	13.97–15.51	1.84	1.84	2.1 mm
Acrylic	1	2.0–2.7	1.59	1.58	2.4 mm

**Table 2:** Parameters for the evaluation of the composite layer system measurement.

Material	Thickness	Measured Thickness BC	Measured Thickness TMM	Optical Correlation Interval	Standard Deviation BC	Standard Deviation TMM
Pertinax	8.04 mm ± 1%	8.08 mm	8.07 mm	(14.5–15.3) mm	2.6 µm	2.2 µm
Acrylic	1.5166 mm ± 0.4%	1.49 mm	1.53 mm	(1.3–2.8) mm	1.4 µm	0 µm (*)

(*) 0.8 µm for the step size of 2.5 µm.

## Supplementary Materials

The following are available online at https://www.mdpi.com/1424-8220/19/18/3910/s1, Figure S1: Color version of solution space of Figure 3.
