# Supplementary figures and images for: Multilayer Thickness Measurements below the Rayleigh Limit Using FMCW Millimeter and Terahertz Waves
Source: Sensors (Basel). 2019 Sep 11;19(18):3910. doi: 10.3390/s19183910 (PMC6767092; doi:10.3390/s19183910)

**Supplementary Materials:**

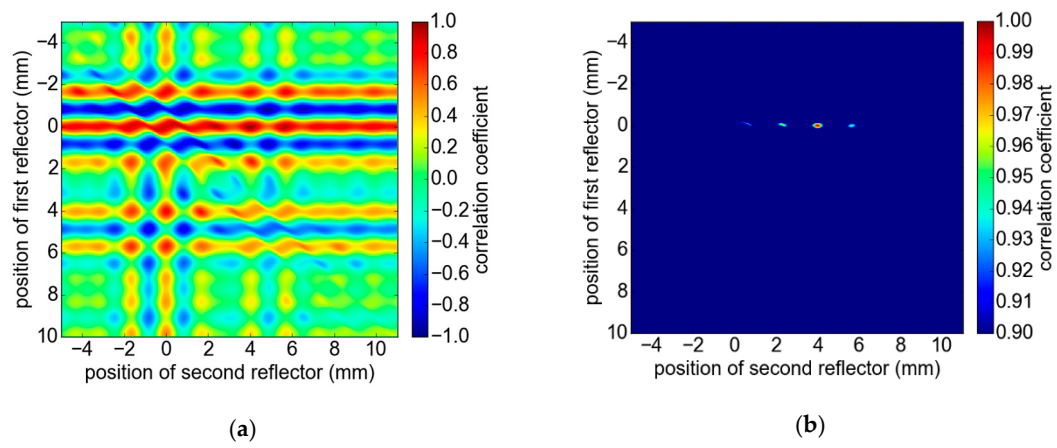

**Figure S1.** Color version of solution space of Figure 3.

Supplement: Supplementary file 1 [file sensors-19-03910-s001.pdf]
